# A rare variation of five major vessels arising from the aortic arch with an absence of brachiocephalic trunk

**DOI:** 10.1007/s00276-025-03674-0

**Published:** 2025-06-19

**Authors:** B. R. Omotoso, R. Harrichandparsad, L. Lazarus

**Affiliations:** 1 Department of Internal Medicine, Faculty of Health Sciences, University of the Free State, Bloemfontein, 9300 South Africa; 2 Discipline of Clinical Anatomy, School of Laboratory Medicine and Medical Sciences, College of Health Sciences, University of KwaZulu-Natal, Westville Campus, Private Bag X54001, Durban, 4000 South Africa; 3 Department of Neurosurgery, School of Clinical Medicine, College of Health Sciences, Nelson R Mandela School of Medicine, University of KwaZulu-Natal, Durban, South Africa

**Keywords:** Vertebral artery, Computed tomography angiography, Brachiocephalic trunk, Aortic arch, Aberrant right subclavian artery

## Abstract

The most common variations of the aortic arch branching pattern usually involve the distance between the vessels arising from it and their dimensions. Changes in the number of vessels originating from the aortic arch, ranging from one to four or more major vessels instead of the classical three vessels as independent branches, are uncommon. Incidents of branching patterns involving four independent vessels arising from the aortic arch are rare, and reports of five or six independent vessels are extremely rare. We report on a case of an absent brachiocephalic trunk associated with an aberrant right subclavian artery and five distinct major vessels arising directly from the aortic arch in a South African male. Although most congenital vascular variations are incidental findings on angiographic images, some have also been associated with cerebrovascular diseases such as cerebral aneurysms. In addition, knowledge of these rare variations is of diagnostic importance as their presence may increase the difficulty and alter the specificity of vascular procedures performed using endovascular and open techniques.

## Introduction

Anatomical variations of the major blood vessels in the head and neck region have been linked with various neurological symptoms, such as ataxia, ischemic strokes, and other cerebrovascular disorders, especially when associated with other vascular diseases, such as aneurysms [3, 19]. However, anatomical variations can be asymptomatic, as most are being discovered as incidental findings during angiographic studies [9, 13]. Whether symptomatic or asymptomatic, detecting variations in the configuration of the aortic arch (AA) branches is extremely important before undertaking procedures or making surgical decisions near the great vessels. According to the classical anatomical description, the brachiocephalic trunk (BCT) is the first branch of the AA. It originates from the convexity of the AA. This is followed by the left common carotid (LCCA) and the left subclavian arteries (LSCA). Complex embryogenesis of the blood vessels of the head and neck region has resulted in various structural congenital variations. The most common variations of the AA branching pattern usually involve the distance between the vessels arising from it and their dimensions. Changes in the number of vessels originating from the AA, ranging from one to four or more major vessels instead of the classical three vessels as independent branches, are uncommon [7, 13]. Therefore, knowledge of the rare branching patterns of the AA is important for surgical, diagnostic, and interventional procedures around the thorax and the neck. The most commonly reported is the so-called bovine aortic arch branching variant (where the LCCA has a common origin with the BCT or originates directly from the BCT) and the direct origin of the left vertebral artery from the AA, both associated with the standard left-sided AA [2]. Other branching patterns involving four independent vessels arising from the AA are rare, and reports of five or six independent vessels are extremely rare [3, 13]. Authors have hypothesized that the incidence of variation in the branching pattern of the AA is high in individuals of African descent [5]. However, reports on the incidence of five independent vessels arising from the AA in the African population are rare [1, 5, 11, 17]. This report presents a case of an absent brachiocephalic trunk associated with an aberrant RSCA and five distinct major vessels arising directly from the AA in a South African male.

## Case presentation

**Fig. 1:**
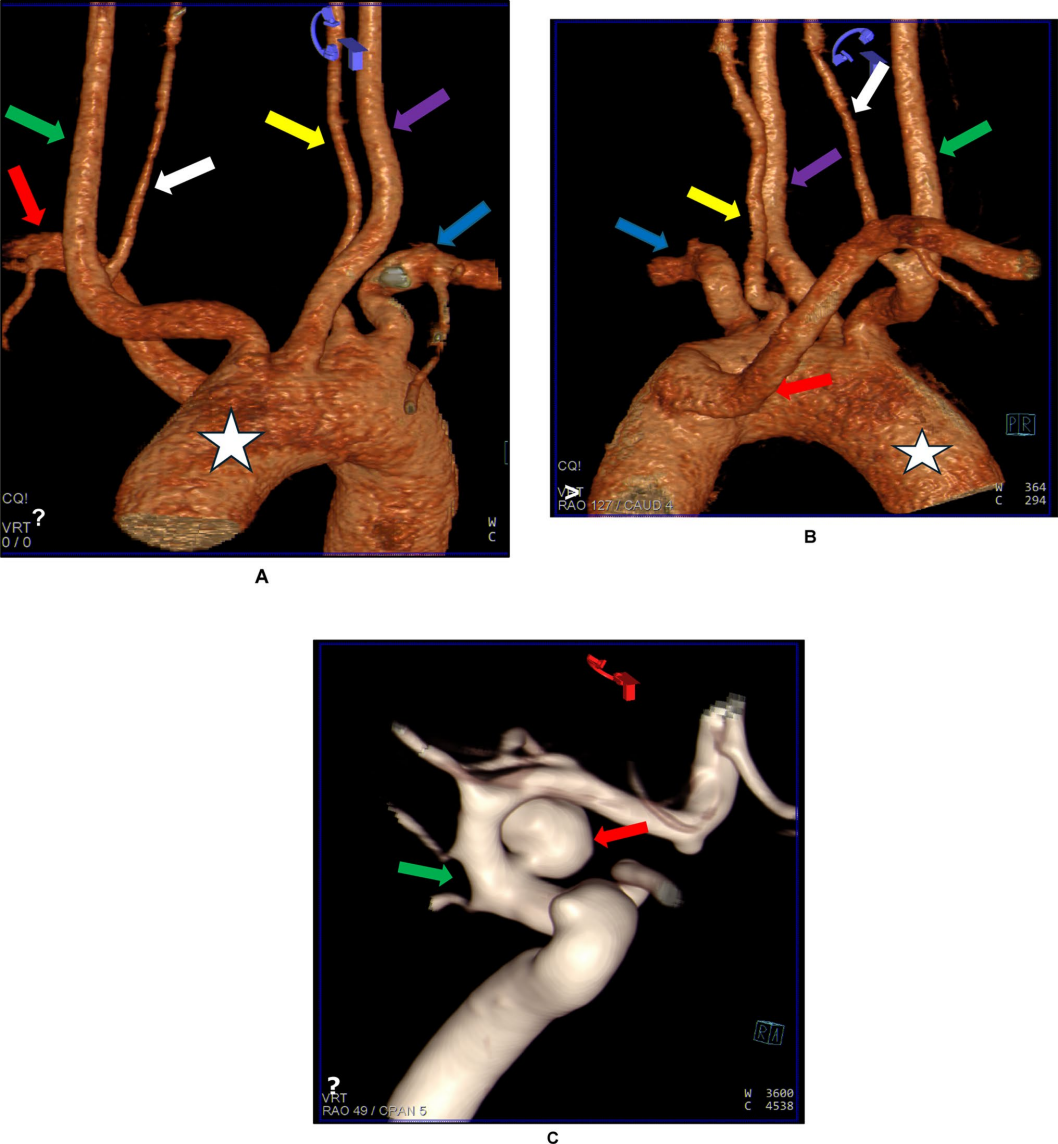
**A** 3D-CTA reconstructed image of the aortic arch (white star) showing the anterolateral view of the arch of the aorta and its branches. An absent brachiocephalic trunk was noticed. The first branch of the arch of the aorta is the right common carotid artery (RCCA-green arrow). The right vertebral artery arises from the anteromedial part of the RCCA (white arrow). The RCCA was followed by the left common carotid artery (LCCA-purple arrow). The left VA directly originates from the arch of the aorta (yellow arrow). The left subclavian artery (LSCA) is the last branch from the anterior view (blue arrow). An aberrant right subclavian artery (RSCA) (arising from the posterior aspect of the arch of the aorta took its origin proximal to the origin of the left subclavian artery (distal portion of the AA) (red arrow). **B** 3D-CTA reconstructed image of the aortic arch (white star) showing the posterior view of the arch of the aorta and its branches. An absent brachiocephalic trunk was noticed. The first branch of the arch of the aorta is the right common carotid artery (RCCA-green arrow). The right vertebral artery arises from the anteromedial part of the RCCA (white arrow). The RCCA was followed by the left common carotid artery (LCCA-purple arrow). The left VA directly originates from the arch of the aorta (yellow arrow). The left subclavian artery (LSCA) is the last branch from the anterior view (blue arrow). An aberrant right subclavian artery (RSCA) (arising from the posterior aspect of the AA) took its origin proximal to the origin of the left subclavian artery (distal portion of the AA) (red arrow). **C** 3D-CTA volume reconstruction of the right ICA run showing the superiorly pointing, posterior communicating artery aneurysm (red arrow) arising from the medial wall of the ICA (green arrow).

A 36-year-old South African male patient presented to the Inkosi Albert Luthuli Central Hospital with a sudden, severe headache and loss of consciousness. The patient had a long-standing and severe smoking history (20 packs per year) with no other vascular risk factors. At presentation to the hospital, his Glasgow Coma Scale (GCS) was 15/15 with no focal neurological deficits (World Federation of Neurological Surgeons (WFNS) grading scale 1). Non-contrast CT brain displayed a thick, diffuse basal subarachnoid hemorrhage extending into the right Sylvian fissure. There was no intraventricular extension (Modified Fisher Grade 3). Volume-reconstructed images of the aortic arch showed a rare aortic arch anatomy. A brachiocephalic trunk was absent, and five distinct major vessels arose directly from the aortic arch in the following order. The first branch was the right common carotid artery (RCCA), followed by the left common carotid artery (LCCA), the left vertebral artery (VA), and the left subclavian trunk (LSCA) (Fig. 1A). Lastly, an aberrant right subclavian artery (RSCA) (which arose from the posterior aspect of the AA) took its origin proximal to the origin of the LSCA (at the distal portion of the AA) from where it crossed to the proximal portion of the AA (Fig. 1B). The right VA arose from the proximal part of the RCCA (Fig. 1A, B). In addition, computed tomography angiography (CTA) showed a right-sided medially and superiorly orientated posterior communicating artery aneurysm arising from the medial wall of the internal carotid artery (Fig. 1C). Following the endovascular intervention to exclude the aneurysm with detachable coils, the patient responded positively with no complications in the post-operative period and was discharged with a GCS 15/15 and had no focal neurological deficits a few days later. The patient will be followed up with a magnetic resonance angiogram in 6 months to exclude aneurysm recanalization. No identifying patient information is present in this paper. The Institutional Research Ethics Committee (University of KwaZulu-Natal Biomedical Research Ethics Committee-Ethical No: BREC/00004897/22) approved this report.

## Discussion

The prevalence of variation in the branching pattern of the AA has been reported in global review studies and meta-analyses [9, 15]. Also, previous large series and case studies have reported variation in the branching pattern of the AA in some African countries, including South Africa [5, 11, 13, 17]. To the best of our knowledge, this is the first report of an absent brachiocephalic trunk associated with an aberrant RSCA and five distinct major vessels arising directly from the AA in a South African population (Table 1). In the present report, the first branch of the AA is the RCCA, followed by the LCCA, the left VA, and LSCA (Fig. 1A). An aberrant right subclavian artery (RSCA) (arising from the posterior aspect of the AA) took its origin proximal to the origin of the left subclavian artery (the distal portion of the AA) from where it crossed to the proximal portion of the AA (Fig. 1B). The right VA arose from the proximal RCCA (Fig. 1A, B). This sequence of branching patterns is rare. In a similar case report from the Sudanese population (cadaveric sample), the left VA arose from the AA between the RCCA and LCCA [1]. In another comparable report from the United States using cadaveric samples, the bilateral vertebral arteries arose directly from the AA, forming the second and the fourth branch in the sequence of five vessel branching patterns of the AA from right to left [7]. The global prevalence of five major vessels arising directly from the AA is approximately 1%; however, this may be present in different configurations of the vessels [9]. An aberrant RSCA has been reported in approximately 0.5 to 1.8% of the population [16]. The incidence of right VA arising from RCCA associated with aberrant RSCA is even rarer, with a prevalence of 0.1% [9]. The most commonly reported variation in the origin of the VA is the origin of the left VA from the AA, which may be as high as 6.9% [5, 9, 14]. The exact cause of variation has not been previously established; however, some authors have hypothesized geographic correlation and the effect of some genetic and environmental factors on the embryogenesis of the supra-aortic arteries and their branches [9, 14, 15, 18].

**Table 1:** The incidence of five independent vessels arising from the aortic arch

Author (Year)	Country	Study type	Sequence of AA branching pattern
Liechty et al. 1957 [5]	USA	Cadaveric	BT, RVA, LCCA, LVA, LSCA
Ma et al. 2017 [19]	China	Cadaveric	RCCA, LCCA, LTT, LSCA, RSCA
Abdelmotalab et al. 2025 [10]	Sudan	Cadaveric	RSCA, RCCA, LVA, LCCA, LSCA
Zaborowski et al. 2025 [20]	Poland	Cadaveric	BT, ITA, LCCA, LVA, LSCA
Present case	South Africa	CTA	RCCA, LCCA, LVA, LSCA, ARSCA

The complex embryogenesis of the AA and its major branches has predisposed the artery to several congenital vascular variations. Variations occur as a result of the persistence of structures that typically completely or incompletely regress [16]. The aortic sac is the first portion of the aorta to develop; it later forms a right and a left horn. The right horn forms the BCT, while the left horn, together with the stem of the aortic sac, forms part of the AA proximal to the BCT [16]. Incomplete development or regression of the right horn may have resulted in the omission of the brachiocephalic trunk and, consequently, the direct origin of the RSCA and RCCA from the AA as reported in the present case (Fig. 1A, B). Another primitive vessel that develops from the aortic sac is the pharyngeal arch arteries (or primitive aortic arches, paired first to sixth). The proximal part of the RSCA develops from the right fourth pharyngeal arch, while the distal part develops from the right seventh cervical intersegmental artery (CIA) (a derivative of the dorsal aorta, which also arises from the aortic sac). We hypothesize that the combination of two different primitive vessels during development may have resulted in the formation of aberrant RSCA crossing from the distal to the proximal portion of the AA in the present report. In contrast, the LSCA develops solely from the left seventh cervical intersegmental artery. Also, the RCCA may have developed from pharyngeal arches other than the third pharyngeal arch, as described by standard anatomy textbooks. The bilateral VA typically develops from longitudinal anastomosis of the right and left seven CIAs. The 1st to 6th CIAs regress to form the segments of the VAs, while the seventh CIA persists in contributing to the formation of the subclavian artery and point of origin of the VA. The persistence of any CIA other than the seventh may lead to variation in the origin and course of the VA [6]. Considering the hypothesis that the persistence of the 6th CIA may result in the origin of the VA from the AA, and the persistence of the 1st or 2nd CIA may be associated with the origin of the VA from the internal or external carotid arteries, as noted in our report, the origin of the right VA from the proximal RCCA may be due to the persistence of either 3rd, 4th or 5th CIA [13].

The functional consequences of the variations in the configuration of the AA branching pattern may include various cerebrovascular abnormalities. For instance, BCT is the first branch of the AA, and it provides blood supply to the right side of the head, neck, and arm. The absence of BCT may reduce the blood flow in the RSCA and RCCA, which typically branch off it, as both have a direct origin from the posterior and the anterior part of the AA, respectively. Thus, the absence of the BCT can reduce blood flow to the right side of the head, neck, and arm. In addition, compromised blood flow in the RCCA may have a direct effect on the right VA that branches off it [13]. The atypical origin of the right VA has been linked to neurological diseases such as ataxia [3], ischemic strokes, and other cerebrovascular disorders [19]. The direct origin of the left VA from the AA has been linked with VA dissection and arteriosclerosis of the first segment [4, 12]. An aberrant RSCA has been associated with symptoms of dyspnoea or dysphagia lusoria and the diverticulum of Kommerell, a bulge in the arch itself [7, 10]. In the present case presentation, the patient was diagnosed with diffused basal subarachnoid hemorrhage and a right-sided supraclinoid aneurysm using non-contrast CT and CTA. Considering the fact that this is an adult patient, the congenital variation in the branching pattern of the AA may not be the direct cause of the subarachnoid hemorrhage and the aneurysm of the posterior communicating artery in the supraclinoid portion of the right internal carotid artery. However, the origin of the right VA from the proximal RCCA, which later divides into the internal and external carotid arteries, may have compromised the dynamics of blood flow in the RCCA tree, generating flow inconsistencies in one of the branches (for instance, the internal carotid artery).

## Limitations

The study reported a case of variation of the aortic arch branching pattern; we cannot generalize the incidence to the entire South African population. The authors are unable to establish the exact cause of the variation.

## Conclusion

The authors describe an extremely rare sequence of five vessels branching pattern of the AA with the absence of a brachiocephalic trunk, an aberrant RSCA, and bilateral variation in the origin of the VAs in a South African male. Although most congenital vascular variations are incidental findings on angiographic images, some have also been associated with cerebrovascular diseases such as cerebral aneurysms. In addition, knowledge of these rare variations is of diagnostic importance as their presence may increase the difficulty and alter the specificity of vascular procedures performed using endovascular and open techniques.

## Data Availability

No datasets were generated or analysed during the current study.
